# Sensor Scheduling for Remote State Estimation with Limited Communication Resources: A Time- and Event-Triggered Hybrid Approach

**DOI:** 10.3390/s23218667

**Published:** 2023-10-24

**Authors:** Yuqing Ni, Xiaochen Liu, Chao Yang

**Affiliations:** 1 The Key Laboratory of Advanced Process Control for Light Industry (Ministry of Education), School of Internet of Things Engineering, Jiangnan University, Wuxi 214122, China; yuqingni@jiangnan.edu.cn; 2 Department of Radiology, Athinoula A. Martinos Center for Biomedical Imaging, Massachusetts General Hospital, Harvard Medical School, Boston, MA 02114, USA; xliu67@mgh.harvard.edu; 3 The Key Laboratory of Smart Manufacturing in Energy Chemical Process (Ministry of Education), Department of Automation, East China University of Science and Technology, Shanghai 200237, China

**Keywords:** remote state estimation, Kalman filtering, sensor scheduling, stochastic event trigger, hybrid scheduling

## Abstract

This paper proposes a time- and event-triggered hybrid scheduling for remote state estimation with limited communication resources. A smart sensor observes a physical process and decides whether to send the local state estimate to a remote estimator via a wireless communication channel; the estimator computes the state estimate of the process according to the received data packets and the known scheduling mechanism. Based on the existing optimal time-triggered scheduling, we employ a stochastic event trigger to save precious communication chances and further improve the estimation performance. The minimum mean-squared error (MMSE) state estimate is derived since the Gaussian property is preserved. The estimation performance upper bound and communication rate are analyzed. The main results are illustrated by numerical examples.

## 1. Introduction

In the last two decades, control systems utilizing wireless networks have seen extensive application due to the rapid and advanced development of sensing, information processing, and communication technologies. They have been extensively applied in industry manufacturing, target tracking and localization, smart grid, automobiles, and unmanned aerial vehicles [1,2,3,4,5].

In practical applications, the majority of sensors rely on battery power, and the replacement of batteries can be both expensive and time-consuming. This issue becomes even more challenging in extreme environments where battery replacement may be impossible. Additionally, the limited bandwidth of wireless communication poses another constraint. Therefore, the scheduling of sensor transmissions becomes a crucial concern in addressing these challenges. One direction is the time-triggered scheduling approach. For example, Savage and La Scala [6] investigated the sensor scheduling for Gauss–Markov systems, and simple index policies were proved to be optimal. Shi et al. [7] studied the two-sensor scheduling problem and constructed an optimal periodic schedule minimizing the estimation error and satisfying the energy and communication bandwidth constraints. Yang and Shi [8] provided an explicit form of the optimal sensor data scheduling with limited communication resources during a finite time horizon. Wang et al. [9] proposed an energy-aware sensor scheduling for target surveillance applications. Usually, such scheduling approaches are well designed in advance, i.e., sensors periodically transmit their data at fixed intervals, regardless of the significance or relevance of the information being transmitted. This can lead to inefficient resource usage, especially when the data being transmitted remain unchanged or are not significant.

In contrast, event-triggered scheduling operates based on the occurrence of specific events or conditions. Instead of transmitting data at fixed intervals, sensors only transmit data when certain predefined events or conditions are met. These events or conditions can be based on thresholds, changes in data values, or other predefined triggers. By transmitting data only when necessary, event-triggered scheduling can significantly reduce the amount of communication required and conserve sensor resources such as battery power and network bandwidth. In the context of remote state estimation, Wu et al. [10] proposed a deterministic event trigger based on the transformed innovation term and an approximate estimator was derived. Han et al. [11] proposed a stochastic event trigger and investigated the state estimation performance. Hu et al. [12] designed a recursive event-triggered state estimation with randomly switching topologies and multiple missing measurements. Wang and Liu [13] further investigated the event-triggered state estimation against denial-of-service (DoS) attacks. Yan et al. [14] proposed a novel adaptive memory-event-triggered mechanism to choose the control signals. For networked nonlinear interconnected systems, Gu et al. [15] provided an adaptive event-triggered scheme and derived the sufficient conditions for stability. Overall, event-triggered scheduling offers a promising approach to optimize the utilization of real-time information, guarantee the system performance, and conserve sensor communication resources. In addition to scheduling, the associated state estimation is also quite important. Sinopoli et al. [16] examined the estimation performance in relation to the measurement packet arrival rate when random packet dropouts occur. Malyavej and Savkin [17] quantized the measurement for a digital communication channel with bit-rate limits and created an encoder and decoder pair to achieve the optimal state estimation via a channel with restricted capacity. Furthermore, Wang et al. [18] investigated the variance-constrained robust estimation in the presence of random delays and packet dropouts in the channel with restricted communication capacity. However, it is worth noting that the communication capacity constraint in the aforementioned works primarily focuses on the transmission bit. In contrast, our work aims to make transmission decisions based on real-time information. Even when the sensor chooses not to transmit, the remote estimator can utilize the sensor’s behavior to extract additional information and enhance estimation performance.

**Figure 1:**
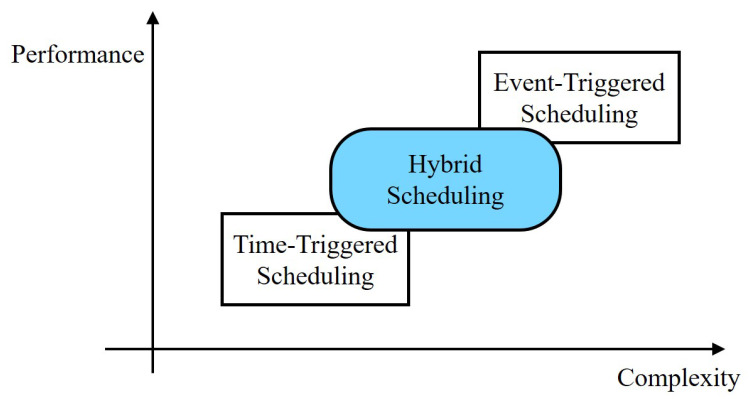
The hybrid scheduling provides a trade-off between the time-triggered scheduling and event-triggered scheduling in terms of computation complexity and state estimation quality.

In this paper, we propose a hybrid time- and event-triggered scheduling approach, which keeps the simplicity of time-triggered scheduling and also further exploits the real-time measurement information to improve communication utilization through the event-triggered mechanism, as shown in Figure 1. Based on the optimal periodic time-triggered scheduling, a stochastic event trigger is employed to check the significance of the measurement and decide whether to transmit it.

The main contributions of this work are summarized as follows.

1. In this work, we develop a hybrid scheduling for remote state estimation with a smart sensor. We combine a stochastic event trigger with an optimal periodic time trigger to enhance the estimation performance while preserving the communication rate.
2. Under the proposed hybrid scheduling, the Gaussian property of the state estimator is preserved, and we derive the MMSE state estimator. To the best of our knowledge, it is the first time that an MMSE state estimator has been developed for the combination of a stochastic event trigger and an optimal periodic time trigger.
3. For the proposed hybrid scheduling, we provide the upper bound of the state estimation error covariance. Moreover, for the stable system, we characterize the transmission probability at the scheduled time, which is decided by the original optimal time trigger. The stochastic transmission implies the communication resource preservation brought by the stochastic event trigger.

The remainder of this paper is organized as follows. Section 2 introduces the setup of the scheduling and remote estimation. The problem of interest is proposed. Section 3 provides the main theoretical results. The time- and event-triggered scheduling is proposed, and its performance is analyzed. Section 4 provides simulations and interpretations. Section 5 draws conclusions.

*Notations*: $Z_{++}$ is the set of positive integers. $k\in Z_{++}$ is the time index. R denotes the set of real numbers. $R^{n}$ is the *n*-dimensional Euclidean space. $S_{+}^{n}$ ($S_{++}^{n}$) is the set of n×n positive semi-definite (definite) matrices. When $X\in S_{+}^{n}$ ($S_{++}^{n}$), we simply write X⪰0 (X≻0). The identity matrix with size *n* is represented by $I_{n}$. The superscript ${}^{⊤}$, Tr{·}, det(·), and $\rho \left(\cdot \right)$ stand for the transpose, trace, determinant, and spectral radius of a matrix, respectively. Pr(·) is the probability of a random variable. E[·] denotes the expectation of a random variable, and E[·|·] denotes the conditional expectation. N(μ,Σ) denotes a Gaussian distribution with the mean μ and covariance Σ. For functions $f,f_{1},$ and $f_{2}$, $f_{1}\circ f_{2}$ is defined as $f_{1}\circ f_{2}\left(X\right)≜f_{1}\left(f_{2}\left(X\right)\right)$ and $f^{k}$ is defined as $f^{k}\left(X\right)≜\underset{k\;\mathrm{times}}{\underset{︸}{f\circ f\circ \cdots \circ f}}\left(X\right)$ with $f^{0}\left(X\right)=X$. For x∈R, ⌊x⌋ denotes the largest integer less than or equal to *x*. The $\ell^{2}$-norm of a vector is denoted by ∥·∥.

## 2. Preliminaries

**Figure 2:**
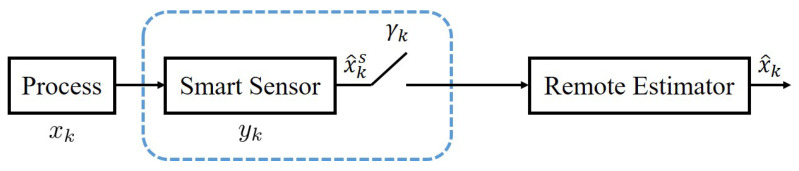
Traditional system diagram.

Consider a system as illustrated in Figure 2. A physical process, a smart sensor, and a remote estimator are involved. In this section, we present the mathematical model of the process and smart sensor. For general scheduling approaches, the estimation process is introduced. The scheduling problem under limited communication resources is provided.

### 2.1. Process and Smart Sensor

A discrete-time linear time-invariant (LTI) process is as follows:

(1) $$\begin{array}{c} x_{k}=Ax_{k-1}+w_{k-1}, \end{array}$$

where $x_{k}\in R^{n}$ is the process state at time *k*, and $w_{k}\in R^{n}$ is an i.i.d. zero-mean Gaussian noise with covariance Q≻0. A smart sensor observes the process and obtains a measurement, $y_{k}$, at time *k*:

(2) $$\begin{array}{c} y_{k}=Cx_{k}+v_{k}, \end{array}$$

where $y_{k}\in R^{m}$ and $v_{k}$ is another i.i.d. zero-mean Gaussian noise with covariance R≻0. The process noise, $w_{k}$, the measurement noise, $v_{k}$, and the initial state, $x_{0}∼N\left(0,\Pi_{0}\right)$, are mutually uncorrelated. The pair $\left(A,Q^{\frac{1}{2}}\right)$ is assumed to be controllable, and (A,C) is observable.

Furthermore, the smart sensor has certain computation capabilities and is capable of running a local Kalman filter based on the collected measurements. Its MMSE state estimate, ${\hat{x}}_{k}^{s}$, and the corresponding error covariance, $P_{k}^{s}$, are denoted as

$$\begin{array}{cc} {\hat{x}}_{k}^{s} & ≜E\left[x_{k}|Y_{k}\right],\\ P_{k}^{s} & ≜E\left[\left(x_{k}-{\hat{x}}_{k}^{s}\right){\left(x_{k}-{\hat{x}}_{k}^{s}\right)}^{⊤}|Y_{k}\right], \end{array}$$

where $Y_{k}≜\left\{y_{1},y_{2},\cdots ,y_{k}\right\}$ is the information set of the smart sensor at time *k*. They are computed via a Kalman filter as follows:

(3) $$\begin{array}{cc} {\hat{x}}_{k|k-1}^{s} & =A{\hat{x}}_{k-1}^{s}, \end{array}$$

(4) $$\begin{array}{cc} P_{k|k-1}^{s} & =AP_{k-1}^{s}A^{⊤}+Q, \end{array}$$

(5) $$\begin{array}{cc} K_{k}^{s} & =P_{k|k-1}^{s}C^{⊤}{\left(CP_{k|k-1}^{s}C^{⊤}+R\right)}^{-1}, \end{array}$$

(6) $$\begin{array}{cc} {\hat{x}}_{k}^{s} & ={\hat{x}}_{k|k-1}^{s}+K_{k}^{s}\left(y_{k}-C{\hat{x}}_{k|k-1}^{s}\right), \end{array}$$

(7) $$\begin{array}{cc} P_{k}^{s} & =\left(I_{n}-K_{k}^{s}C\right)P_{k|k-1}^{s}, \end{array}$$

where ${\hat{x}}_{k|k-1}^{s}$ and $P_{k|k-1}^{s}$ are the a priori state estimate and the associated estimation error covariance. The recursion begins from ${\hat{x}}_{0}^{s}=0$ and $P_{0}^{s}=\Pi_{0}$. From [19], the estimation error covariance $P_{k}^{s}$ of the Kalman filter converges to a unique value, ${\bar{P}}^{s}$, no matter what the initial value is. For presentation brevity, we denote the operators $h,g:S_{+}^{n}\mapsto S_{+}^{n}$ as

$$\begin{array}{cc} h(X) & ≜AXA^{⊤}+Q,\\ g\left(X\right) & ≜X-XC^{⊤}{\left(CXC^{⊤}+R\right)}^{-1}CX. \end{array}$$

We assume that both the a priori and a posteriori error covariances at the smart sensor have already reached the steady states, and let

$$\begin{array}{c} P_{k}^{s}={\bar{P}}^{s},\;\;\;P_{k|k-1}^{s}=P^{s},\;\;\;k\in Z_{++}, \end{array}$$

where ${\bar{P}}^{s}$ is the unique positive semi-definite solution to g∘h(X)=X and $P^{s}$ is the unique positive semi-definite solution to h∘g(X)=X. This results in a steady-state local Kalman filter with a fixed gain, $K^{s}=P^{s}C^{⊤}{\left(CP^{s}C^{⊤}+R\right)}^{-1}$.

### 2.2. Transmission with Limited Communication Resources

The smart sensor transmits its local estimate, ${\hat{x}}_{k}^{s}$, to the remote estimator via a wireless communication channel. However, in the implementation of a wireless network, sensors are often impossible to recharge; meanwhile, the data transmission usually consumes most of the sensor power. Hence, the transmission chances of the sensors are limited and scarce. We assume that the smart sensor has, at most, $d\in Z_{++}$ times available to communicate with the remote estimator during a finite time horizon, $T\in Z_{++}$, where T>>d. The smart sensor needs to decide at which time instances to transmit the local estimates. The indicator variable $\gamma_{k}$ is used to denote the transmission. If the local state estimate, ${\hat{x}}_{k}^{s}$, is transmitted at time *k*, $\gamma_{k}=1$; otherwise, $\gamma_{k}=0$.

### 2.3. Remote Estimator

Based on the received data packets, the remote estimator computes the MMSE state estimate, ${\hat{x}}_{k}$, and this and the corresponding estimation error covariance $P_{k}$ are defined as

$$\begin{array}{cc} {\hat{x}}_{k} & ≜E\left[x_{k}|I_{k}\right],\\ P_{k} & ≜E\left[\left(x_{k}-{\hat{x}}_{k}\right){\left(x_{k}-{\hat{x}}_{k}\right)}^{⊤}|I_{k}\right], \end{array}$$

where $I_{k}≜\left\{\gamma_{1},\gamma_{2},\ldots ,\gamma_{k},\gamma_{1}{\hat{x}}_{1}^{s},\gamma_{2}{\hat{x}}_{2}^{s},\ldots ,\gamma_{k}{\hat{x}}_{k}^{s}\right\}$ is the information set of the remote estimator at time *k*. They follow the recursion [20]:

(8) $$\begin{array}{cc} {\hat{x}}_{k} & =\left\{\begin{array}{cc} {\hat{x}}_{i}^{s}, & \;\mathrm{if}\;\gamma_{k}=1,\\ A{\hat{x}}_{k-1},\; & \;\mathrm{if}\;\gamma_{k}=0, \end{array}\right. \end{array}$$

(9) $$\begin{array}{cc} P_{k} & =\left\{\begin{array}{cc} {\bar{P}}^{s}, & \;\mathrm{if}\;\gamma_{k}=1,\\ h(P_{k-1}),\; & \;\mathrm{if}\;\gamma_{k}=0. \end{array}\right. \end{array}$$

The initial values are ${\hat{x}}_{0}=0$ and $P_{0}={\bar{P}}^{s}$.

### 2.4. Problem of Interest

We consider a sensor scheduling problem in the context of remote state estimation. The purpose is to minimize the average state estimation error covariance during a finite time horizon, as follows:

(10) $$\begin{array}{c} J≜\frac{1}{T}\sum_{k=1}^{T}\mathrm{Tr}\left\{E\left[P_{k}\right]\right\}. \end{array}$$

We aim to propose a time- and event-triggered hybrid sensor scheduling policy under the communication resource constraint $\sum_{k=1}^{T}\gamma_{k}\leq d$.

## 3. Time- and Event-Triggered Hybrid Sensor Scheduling

In this section, we attempt to sufficiently utilize the limited communication chances to optimize the estimation performance of the remote estimator. First, we present the optimal pure time-triggered scheduling and then the stochastic event-triggered mechanism. Finally, we combine both scheduling policies and propose a hybrid approach to further improve the estimation performance.

### 3.1. Optimal Time-Triggered Scheduling

Before providing the optimal time-triggered scheduling, we define some necessary variables and parameters. For each transmission policy $\left\{\gamma_{k}\right\}$ satisfying $\sum_{k=1}^{T}\gamma_{k}=d$, we define a transmission time index sequence $\left\{k_{1},k_{2},\ldots ,k_{d}\right\}$, where $1\leq k_{1}<k_{2}<\cdots <k_{d}\leq T$ and $k_{i}$ is the time instance when $\gamma_{k_{i}}=1,\;1\leq i\leq d$. Let $\omega_{i}≜k_{i}-k_{i-1},\;2\leq i\leq d$, and $\omega_{1}≜k_{1}$, $\omega_{d+1}≜T+1-k_{d}$. Define $M≜\lfloor \frac{T+1}{d+1}\rfloor$ and N≜(T+1)mod(d+1). The optimal time-triggered scheduling is presented by the following theorem [21,22].

**Lemma 1.**  *A necessary and sufficient condition for the scheduling policy $\left\{\gamma_{k}^{★}\right\}$ to be optimal is that $\sum_{k=1}^{T}\gamma_{k}^{★}=d$ and the associated $\omega_{i}^{★}$’s satisfy*

$$\begin{array}{cc} \omega_{i}^{★}=M\;\mathrm{or}\;M+1, & \;\;i=1,2,\cdots ,d+1. \end{array}$$

*Furthermore, among all the $\omega_{i}^{★}$’s, N of them are equal to M+1, and d+1−N of them are equal to M. The corresponding state estimation error covariance is*

$$\begin{array}{c} J^{★}=\frac{1}{T}\mathrm{Tr}\left\{d{\bar{P}}^{s}+\left(d+1\right)\sum_{i=1}^{M-1}h^{i}\left({\bar{P}}^{s}\right)+Nh^{M}\left({\bar{P}}^{s}\right)\right\}. \end{array}$$

**Proof**  (Proof of Lemma 1). See Theorem 3.2 in [22].    □

**Remark 1.**  *The intuitive representation of Lemma 1 is that the transmission times of the sensor should be distributed as uniformly as possible.*

### 3.2. Stochastic Event-Triggered Scheduling

**Figure 3:**
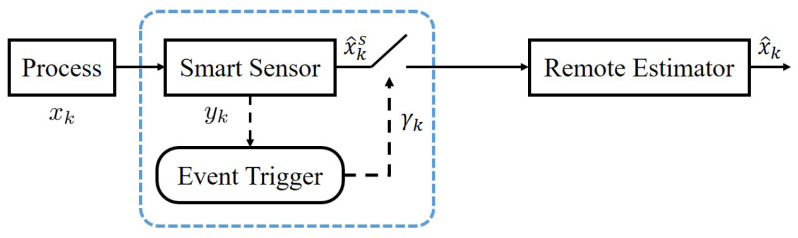
Scheduling with an event trigger.

The time-triggered scheduling policy is offline, which means it is designed before the process operation and consequently is unable to use real-time information. We attempt to employ an online scheduling policy where the smart sensor follows a stochastic decision rule, aiming to further save communication resources. Before combining both scheduling policies, we introduce the real-time event-triggered scheduling policy, as shown in Figure 3.

Generally, the event-triggered scheduling means that the smart sensor transmits the data packet only when a triggering condition is satisfied. For example, $∥y_{k}-b∥\leq \epsilon$, where $b\in R^{n}$ and ϵ∈R are pre-specified, is a usually deployed condition. Under this event-triggered mechanism, when the condition is met at time *k*, the smart sensor does not need to transmit ${\hat{x}}_{k}^{s}$, which saves the communication resource. Meanwhile, although not receiving ${\hat{x}}_{k}^{s}$, the remote estimator knows that $y_{k}$ is around the value *b* and could compute a better state estimate rather than simply carrying out the time update $A{\hat{x}}_{k-1}^{s}$. This is called a deterministic trigger. However, these kinds of triggering conditions cause a truncated probability density function at the remote estimator side [10,21,23], and the MMSE state estimates cannot preserve the Gaussian property anymore. Consequently, the Kalman filtering procedure cannot provide an MMSE estimation performance.

To address this problem, we propose a stochastic event trigger. In the stochastic event-triggered mechanism, the smart sensor generates an i.i.d. random variable, $\zeta_{k}\in R$, following a uniform distribution over [0,1] at time *k*. Then, the smart sensor compares $\zeta_{k}$ with the value of $\phi (y_{k})$. The function $\phi :R^{m}\mapsto R$, and $\phi (y_{k})$ is given by

(11) $$\begin{array}{c} \phi \left(y_{k}\right)=\exp\left(-\frac{1}{2}{\left(y_{k}-b\right)}^{⊤}Y\left(y_{k}-b\right)\right), \end{array}$$

where $b\in R^{m}$ and $Y\in S_{++}^{m}$ are given parameters. The triggering condition is described as follows:

(12) $$\begin{array}{c} \gamma_{k}=\left\{\begin{array}{cc} 0, & \;\mathrm{if}\;\zeta_{k}\leq \phi \left(y_{k}\right),\\ 1, & \;\mathrm{if}\;\zeta_{k}>\phi \left(y_{k}\right). \end{array}\right. \end{array}$$

**Remark 2**  (The stochastic event trigger). *The triggering condition $\zeta_{k}\leq \phi \left(y_{k}\right)$ is stochastic. Similar event triggers are proposed in [11,24]. This trigger has the same purpose as the truncated one: if the value of $y_{k}$ is closer to b, the result of $\phi (y_{k})$ is closer to 1, and consequently, the condition $\zeta_{k}\leq \phi \left(y_{k}\right)$ is more likely to be triggered. The intuitive interpretation of the mechanism is that once the estimator does not receive any data packet at the pre-scheduled time instance k, it knows that $y_{k}$ is close to b. Compared with a deterministic truncated trigger such as $∥y_{k}-b∥\leq \epsilon$, the advantage of using the stochastic trigger is that it simplifies the estimation procedure: it preserves the Gaussian property of the state estimator, which can be found in the succeeding theorem.*

Before presenting the main result, we define ${\hat{x}}_{k|k-1}≜E\left[x_{k}|I_{k-1}\right]$ and $P_{k|k-1}≜E\left[\left(x_{k}-{\hat{x}}_{k|k-1}\right){\left(x_{k}-{\hat{x}}_{k|k-1}\right)}^{⊤}|I_{k-1}\right]$ as the a priori state estimate and the associated estimation error covariance. Let ${\hat{y}}_{k|k-1}≜C{\hat{x}}_{k|k-1}$. The following lemma is also needed, and its proof can be found in the Appendix A.

**Lemma 2.**  *For block matrices $\Phi \in S_{++}^{n+m}$ and $\Theta \in S_{++}^{n+m}$ being*

$$\begin{array}{c} \Phi ≜\left[\begin{array}{cc} \Phi_{xx} & \Phi_{xy}\\ \Phi_{yx} & \Phi_{yy} \end{array}\right],\;\;\;\Theta ≜\left[\begin{array}{cc} \Theta_{xx} & \Theta_{xy}\\ \Theta_{yx} & \Theta_{yy} \end{array}\right], \end{array}$$

*where $\Phi_{xx},\Theta_{xx}\in S_{++}^{n}$, $\Phi_{yy},\Theta_{yy}\in S_{++}^{m}$, and $\Phi_{xy}=\Phi_{yx}^{⊤},\;\Theta_{xy}=\Theta_{yx}^{⊤}$, we let*

(13) $$\begin{array}{c} \Theta^{-1}=\Phi^{-1}+\left[\begin{array}{cc} 0 & 0\\ 0 & Y \end{array}\right]. \end{array}$$

*Then, we have*

(14) $$\begin{array}{cc} \Theta_{xx} & =\Phi_{xx}-\Phi_{xy}{\left(\Phi_{yy}+Y^{-1}\right)}^{-1}\Phi_{yx}, \end{array}$$

(15) $$\begin{array}{cc} \Theta_{xy} & =\Phi_{xy}{\left(I_{m}+Y\Phi_{yy}\right)}^{-1}, \end{array}$$

(16) $$\begin{array}{cc} \Theta_{yy} & ={\left(\Phi_{yy}^{-1}+Y\right)}^{-1}. \end{array}$$

**Theorem 1**  (Virtual Sensor). *When $\gamma_{k}=0$ with a stochastic event trigger *(12)*, at the remote estimator side, $x_{k}$ conditioned on $I_{k-1}$ follows the Gaussian distribution $N\left({\hat{x}}_{k|k-1},P_{k|k-1}\right)$, while $x_{k}$ conditioned on $I_{k}$ follows the Gaussian distribution $N\left({\hat{x}}_{k},P_{k}\right)$. It is equivalent for the estimator to receive ${\tilde{y}}_{k}$ from a virtual sensor, where*

(17) $$\begin{array}{c} {\tilde{y}}_{k}=Cx_{k}+{\tilde{v}}_{k},\;\;\;{\tilde{v}}_{k}∼N\left(0,R+Y^{-1}\right), \end{array}$$

*and ${\tilde{y}}_{k}=b$. Correspondingly, the MMSE estimator computes ${\hat{x}}_{k}$ and $P_{k}$ as follows:*

(18) $$\begin{array}{cc} {\hat{x}}_{k|k-1} & =A{\hat{x}}_{k-1}, \end{array}$$

(19) $$\begin{array}{cc} P_{k|k-1} & =h\left(P_{k-1}\right), \end{array}$$

(20) $$\begin{array}{cc} K_{k} & =P_{k|k-1}C^{⊤}{\left(CP_{k|k-1}C^{⊤}+R+Y^{-1}\right)}^{-1}, \end{array}$$

(21) $$\begin{array}{cc} {\hat{x}}_{k} & ={\hat{x}}_{k|k-1}+K_{k}\left(b-C{\hat{x}}_{k|k-1}\right), \end{array}$$

(22) $$\begin{array}{cc} P_{k} & =\left(I_{n}-K_{k}C\right)P_{k|k-1}. \end{array}$$

**Proof** **ofTheorem** **1.** We prove the argument by mathematical induction. For k=1, since $I_{0}=⌀$, $x_{1}$ conditioned on $I_{0}$ follows $N\left(0,h\left(P_{0}\right)\right)$, we first focus on the a posteriori estimation process. We assume that for k=t, $x_{t}$ conditioned on $I_{t-1}$ follows $N\left({\hat{x}}_{t|t-1},P_{t|t-1}\right)$, where ${\hat{x}}_{t|t-1}=A{\hat{x}}_{t-1}$ and $P_{t|t-1}=h\left(P_{t-1}\right)$. Then, the joint conditional probability density function of $x_{t}$ and $y_{t}$ given $I_{t}$ is

(23) $$\begin{array}{cc} f\left(x_{t},y_{t}|I_{t}\right) & =f\left(x_{t},y_{t}|\gamma_{t}=0,I_{t-1}\right)\\ \; & =\frac{\mathrm{Pr}\left(\gamma_{t}=0|x_{t},y_{t},I_{t-1}\right)f\left(x_{t},y_{t}|I_{t-1}\right)}{\mathrm{Pr}\left(\gamma_{t}=0|I_{t-1}\right)}\\ \; & =\frac{\mathrm{Pr}\left(\gamma_{t}=0|y_{t}\right)f\left(x_{t},y_{t}|I_{t-1}\right)}{\mathrm{Pr}\left(\gamma_{t}=0|I_{t-1}\right)}\\ \; & =\frac{1}{\mathrm{Pr}\left(\gamma_{t}=0|I_{t-1}\right)\sqrt{\det\left(\Phi_{t}\right){\left(2\pi \right)}^{m+n}}}\exp\left(-\frac{1}{2}\theta_{t}\right), \end{array}$$

where $\Phi_{t}$ is the covariance of ${\left[x_{t}^{⊤},\;y_{t}^{⊤}\right]}^{⊤}$ given $I_{k-1}$:

$$\begin{array}{cc} \Phi_{t} & ≜\left[\begin{array}{cc} \Phi_{t,xx} & \Phi_{t,xy}\\ \Phi_{t,yx} & \Phi_{t,yy} \end{array}\right]=\left[\begin{array}{cc} P_{t|t-1} & P_{t|t-1}C^{⊤}\\ CP_{t|t-1} & CP_{t|t-1}C^{⊤}+R \end{array}\right], \end{array}$$

and

(24) $$\begin{array}{cc} \theta_{t} & ≜{\left[\begin{array}{c} x_{t}-{\hat{x}}_{t|t-1}\\ y_{t}-{\hat{y}}_{t|t-1} \end{array}\right]}^{⊤}\Phi_{t}^{-1}\left[\begin{array}{c} x_{t}-{\hat{x}}_{t|t-1}\\ y_{t}-{\hat{y}}_{t|t-1} \end{array}\right]+{\left(y_{t}-b\right)}^{⊤}Y\left(y_{t}-b\right). \end{array}$$

In Equation (23), the second equality holds due to the Bayes’ theorem, and the third equality holds because $\gamma_{t}$ is conditionally independent of $x_{t}$ and $I_{t-1}$ given $y_{t}$. The last equality is directly derived from the induction assumption and the stochastic event trigger condition (12). For $\theta_{t}$ in Equation (24), we aim to put the $y_{t}$’s together using the following procedure. Define $\Theta_{t}≜\left[\begin{array}{cc} \Theta_{t,xx} & \Theta_{t,xy}\\ \Theta_{t,yx} & \Theta_{t,yy} \end{array}\right]={\left(\Phi_{t}^{-1}+\left[\begin{array}{cc} 0 & 0\\ 0 & Y \end{array}\right]\right)}^{-1}$. Then, it is re-arranged mainly by completing the square.

(25) $$\begin{array}{cc} \theta_{t} & ={\left[\begin{array}{c} x_{t}-{\hat{x}}_{t|t-1}\\ y_{t}-{\hat{y}}_{t|t-1} \end{array}\right]}^{⊤}\Phi_{t}^{-1}\left[\begin{array}{c} x_{t}-{\hat{x}}_{t|t-1}\\ y_{t}-{\hat{y}}_{t|t-1} \end{array}\right]+{\left[\begin{array}{c} x_{t}\\ y_{t}-b \end{array}\right]}^{⊤}\left[\begin{array}{cc} 0 & 0\\ 0 & Y \end{array}\right]\left[\begin{array}{c} x_{t}\\ y_{t}-b \end{array}\right]\\ \; & ={\left[\begin{array}{c} x_{t}\\ y_{t} \end{array}\right]}^{⊤}\left(\Phi_{t}^{-1}+\left[\begin{array}{cc} 0 & 0\\ 0 & Y \end{array}\right]\right)\left[\begin{array}{c} x_{t}\\ y_{t} \end{array}\right]-2\left({\left[\begin{array}{c} {\hat{x}}_{t|t-1}\\ {\hat{y}}_{t|t-1} \end{array}\right]}^{⊤}\Phi_{t}^{-1}+{\left[\begin{array}{c} 0\\ b \end{array}\right]}^{⊤}\left[\begin{array}{cc} 0 & 0\\ 0 & Y \end{array}\right]\right)\left[\begin{array}{c} x_{t}\\ y_{t} \end{array}\right]+{\left[\begin{array}{c} {\hat{x}}_{t|t-1}\\ {\hat{y}}_{t|t-1} \end{array}\right]}^{⊤}\Phi_{t}^{-1}\left[\begin{array}{c} {\hat{x}}_{t|t-1}\\ {\hat{y}}_{t|t-1} \end{array}\right]+{\left[\begin{array}{c} 0\\ b \end{array}\right]}^{⊤}\left[\begin{array}{cc} 0 & 0\\ 0 & Y \end{array}\right]\left[\begin{array}{c} 0\\ b \end{array}\right]\\ \; & ={\left[\left[\begin{array}{c} x_{t}\\ y_{t} \end{array}\right]-\Theta_{t}\left(\Phi_{t}^{-1}\left[\begin{array}{c} {\hat{x}}_{t|t-1}\\ {\hat{y}}_{t|t-1} \end{array}\right]+\left[\begin{array}{c} 0\\ Yb \end{array}\right]\right)\right]}^{⊤}\Theta_{t}^{-1}\left[\left[\begin{array}{c} x_{t}\\ y_{t} \end{array}\right]-\Theta_{t}\left(\Phi_{t}^{-1}\left[\begin{array}{c} {\hat{x}}_{t|t-1}\\ {\hat{y}}_{t|t-1} \end{array}\right]+\left[\begin{array}{c} 0\\ Yb \end{array}\right]\right)\right]+{\left[\begin{array}{c} {\hat{x}}_{t|t-1}\\ {\hat{y}}_{t|t-1} \end{array}\right]}^{⊤}\Phi_{t}^{-1}\left[\begin{array}{c} {\hat{x}}_{t|t-1}\\ {\hat{y}}_{t|t-1} \end{array}\right]+b^{⊤}Yb\\ \; & \;\;\;\;\;-{\left(\Phi_{t}^{-1}\left[\begin{array}{c} {\hat{x}}_{t|t-1}\\ {\hat{y}}_{t|t-1} \end{array}\right]+\left[\begin{array}{c} 0\\ Yb \end{array}\right]\right)}^{⊤}\Theta_{t}\left(\Phi_{t}^{-1}\left[\begin{array}{c} {\hat{x}}_{t|t-1}\\ {\hat{y}}_{t|t-1} \end{array}\right]+\left[\begin{array}{c} 0\\ Yb \end{array}\right]\right). \end{array}$$

For notation brevity, we define

(26) $$\begin{array}{cc} \left[\begin{array}{c} {\bar{x}}_{t}\\ {\bar{y}}_{t} \end{array}\right] & ≜\Theta_{t}\left(\Phi_{t}^{-1}\left[\begin{array}{c} {\hat{x}}_{t|t-1}\\ {\hat{y}}_{t|t-1} \end{array}\right]+\left[\begin{array}{c} 0\\ Yb \end{array}\right]\right), \end{array}$$

(27) $$\begin{array}{cc} c_{t} & ≜{\left[\begin{array}{c} {\hat{x}}_{t|t-1}\\ {\hat{y}}_{t|t-1} \end{array}\right]}^{⊤}\Phi_{t}^{-1}\left[\begin{array}{c} {\hat{x}}_{t|t-1}\\ {\hat{y}}_{t|t-1} \end{array}\right]+b^{⊤}Yb-{\left(\Phi_{t}^{-1}\left[\begin{array}{c} {\hat{x}}_{t|t-1}\\ {\hat{y}}_{t|t-1} \end{array}\right]+\left[\begin{array}{c} 0\\ Yb \end{array}\right]\right)}^{⊤}\Theta_{t}\left(\Phi_{t}^{-1}\left[\begin{array}{c} {\hat{x}}_{t|t-1}\\ {\hat{y}}_{t|t-1} \end{array}\right]+\left[\begin{array}{c} 0\\ Yb \end{array}\right]\right). \end{array}$$

To further simplify the above variables, we have

(28) $$\begin{array}{cc} \Theta_{t}\Phi_{t}^{-1} & =\Theta_{t}\left(\Theta_{t}^{-1}-\left[\begin{array}{cc} 0 & 0\\ 0 & Y \end{array}\right]\right)=I_{n+m}-\left[\begin{array}{cc} 0 & \Theta_{t,xy}Y\\ 0 & \Theta_{t,yy}Y \end{array}\right]=\left[\begin{array}{cc} I_{n} & -\Theta_{t,xy}Y\\ 0 & I_{m}-\Theta_{t,yy}Y \end{array}\right], \end{array}$$

(29) $$\begin{array}{cc} \Phi_{t}^{-1}-\Phi_{t}^{-1}\Theta_{t}\Phi_{t}^{-1} & =\Phi_{t}^{-1}-\Phi_{t}^{-1}{\left(\Phi_{t}^{-1}+\left[\begin{array}{cc} 0 & 0\\ 0 & Y \end{array}\right]\right)}^{-1}\Phi_{t}^{-1}=\left[\begin{array}{cc} 0 & 0\\ 0 & {\left(\Phi_{t,yy}+Y^{-1}\right)}^{-1} \end{array}\right], \end{array}$$

where Equation (29) is derived by the direct product computing of block matrices, as stated in the proof of Lemma 2. Then, based on Lemma 2, Equations (26) and (27) can be re-written as

(30) $$\begin{array}{cc} {\bar{x}}_{t} & ={\hat{x}}_{t|t-1}-P_{t|t-1}C^{⊤}{\left(Y^{-1}+CP_{t|t-1}C^{⊤}+R\right)}^{-1}\left({\hat{y}}_{t|t-1}-b\right), \end{array}$$

(31) $$\begin{array}{cc} {\bar{y}}_{t} & ={\hat{y}}_{t|t-1}-{\left[{\left(CP_{t|t-1}C^{⊤}+R\right)}^{-1}+Y\right]}^{-1}Y\left({\hat{y}}_{t|t-1}-b\right), \end{array}$$

(32) $$\begin{array}{cc} c_{t} & ={\hat{y}}_{t|t-1}^{⊤}{\left(\Phi_{t,yy}+Y^{-1}\right)}^{-1}{\hat{y}}_{t|t-1}+b^{⊤}\left(Y-Y\Theta_{t,yy}Y\right)b-2b^{⊤}\left(Y-Y\Theta_{t,yy}Y\right){\hat{y}}_{t|t-1}\\ & ={\left({\hat{y}}_{t|t-1}-b\right)}^{⊤}{\left(CP_{t|t-1}C^{⊤}+R+Y^{-1}\right)}^{-1}\left({\hat{y}}_{t|t-1}-b\right), \end{array}$$

where Equation (32) is obtained by the matrix inversion lemma. Recalling Equation (25) and Lemma 2, we have

$$\begin{array}{c} \theta_{t}={\left(\left[\begin{array}{c} x_{t}\\ y_{t} \end{array}\right]-\left[\begin{array}{c} {\bar{x}}_{t}\\ {\bar{y}}_{t} \end{array}\right]\right)}^{⊤}\Theta_{t}^{-1}\left(\left[\begin{array}{c} x_{t}\\ y_{t} \end{array}\right]-\left[\begin{array}{c} {\bar{x}}_{t}\\ {\bar{y}}_{t} \end{array}\right]\right)+c_{t}, \end{array}$$

where

$$\begin{array}{cc} \Theta_{t,xx} & =P_{t|t-1}-P_{t|t-1}C^{⊤}{\left(CP_{t|t-1}C^{⊤}+R+Y^{-1}\right)}^{-1}CP_{t|t-1},\\ \Theta_{t,xy} & =P_{t|t-1}C^{⊤}{\left[I_{m}+Y\left(CP_{t|t-1}C^{⊤}+R\right)\right]}^{-1},\\ \Theta_{t,yy} & ={\left[{\left(CP_{t|t-1}C^{⊤}+R\right)}^{-1}+Y\right]}^{-1}. \end{array}$$

Hence, the joint conditional probability density function of $x_{t}$ and $y_{t}$ given $I_{t}$ is

$$\begin{array}{cc} f\left(x_{t},y_{t}|I_{t}\right)= & \frac{1}{\mathrm{Pr}\left(\gamma_{t}=0|I_{t-1}\right)\sqrt{\det\left(\Phi_{t}\right){\left(2\pi \right)}^{m+n}}}\exp\left(-\frac{1}{2}c_{t}\right)\\ \; & \times \exp\left(-\frac{1}{2}{\left(\left[\begin{array}{c} x_{t}\\ y_{t} \end{array}\right]-\left[\begin{array}{c} {\bar{x}}_{t}\\ {\bar{y}}_{t} \end{array}\right]\right)}^{⊤}\Theta_{t}^{-1}\left(\left[\begin{array}{c} x_{t}\\ y_{t} \end{array}\right]-\left[\begin{array}{c} {\bar{x}}_{t}\\ {\bar{y}}_{t} \end{array}\right]\right)\right). \end{array}$$

Due to the definition of a probability density function, i.e., its integral equals one, we obtain

$$\begin{array}{c} \frac{1}{\mathrm{Pr}\left(\gamma_{t}=0|I_{t-1}\right)\sqrt{\det\left(\Phi_{t}\right){\left(2\pi \right)}^{m+n}}}\exp\left(-\frac{1}{2}c_{t}\right)=\frac{1}{\sqrt{\det\left(\Theta_{t}\right){\left(2\pi \right)}^{m+n}}}, \end{array}$$

which implies that $x_{t}$ and $y_{t}$ are jointly Gaussian conditioned on $I_{t}$, $x_{t}|I_{t}∼N\left(\bar{x},\;\Theta_{t,xx}\right)$, and $y_{t}|I_{t}∼N\left(\bar{y},\;\Theta_{t,yy}\right)$. Then, we can conclude that $x_{t}$ conditioned on $I_{t}$ follows a Gaussian distribution, $N\left({\hat{x}}_{t},\;P_{t}\right)$, as Equations (21) and (22) show.

Finally, we consider the a priori estimation process. According to the mathematical induction procedure, we assume that for k=t, $x_{t}$ conditioned on $I_{t}$ follows $N\left({\hat{x}}_{t},\;P_{t}\right)$. Then, $f\left(x_{t+1}|I_{t}\right)=f\left(Ax_{t}+w_{t}|I_{t}\right)$ and $x_{t+1}|I_{t}∼N\left(A{\hat{x}}_{t},\;h\left(P_{t}\right)\right)$, which completes the proof.    □

**Remark 3.**  *Theorem 1 reveals that although the smart sensor does not transmit ${\hat{x}}_{k}^{s}$ at time k, the remote estimator could compute a more accurate state estimate via Equation *(21)* instead of the a priori state estimate with the proposed stochastic event-triggered scheduling.*

### 3.3. Hybrid Scheduling

**Figure 4:**
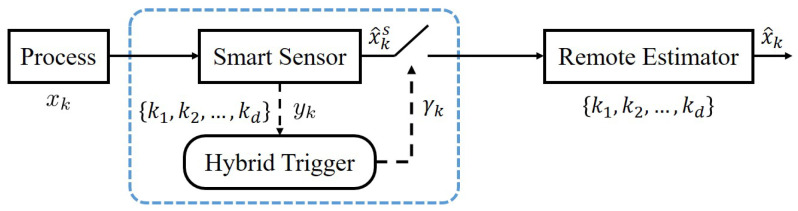
Hybrid scheduling diagram.

In this subsection, we provide a time- and event-triggered hybrid approach for the sensor scheduling problem, which is illustrated in Figure 4 and summarized in Algorithm 1.
**Algorithm 1** Time- and Event-Triggered Hybrid Sensor Scheduling and Estimation1:Input: A,C,Q,R,T,d,Y,b;2:Initialisation: Smart sensor and estimator compute an optimal time-triggered scheduling according to Lemma 1 by following the same pre-specified rule and obtain transmission time index sequence $S≜\left\{k_{1},k_{2},\ldots ,k_{d}\right\}$, respectively. At remote estimator side, ${\hat{x}}_{0}=0$, $P_{0}={\bar{P}}^{s}$;3:**for** k=1:1:T **do**4:   **if** k+d≥T+1 **then**5:     For the remaining time k:1:T, smart sensor transmits ${\hat{x}}_{k}^{s}$, i.e., $\gamma_{k}=1$;6:     For the remaining time k:1:T, estimator updates estimate and covariance by
$$\begin{array}{l} {\hat{x}}_{k}={\hat{x}}_{k}^{s},\\ P_{k}={\bar{P}}^{s}; \end{array}$$7:   **end if**8:   **if** k∉S **then**9:     Smart sensor does not transmit ${\hat{x}}_{k}^{s}$, i.e., $\gamma_{k}=0$;10:     Estimator updates estimate and covariance by
$$\begin{array}{l} {\hat{x}}_{k}=A{\hat{x}}_{k-1},\\ P_{k}=h\left(P_{k-1}\right); \end{array}$$11:   **else**12:     Smart sensor obtains $\zeta_{k}$ from a uniform distribution over $\left[0,\;1\right]$;13:     **if** $\zeta_{k}>\phi \left(y_{k}\right)$ **then**14:        Smart sensor transmits ${\hat{x}}_{k}^{s}$, i.e., $\gamma_{k}=1$;15:        Smart sensor and estimator update d←d−1;16:        Estimator updates estimate and covariance by
$$\begin{array}{l} {\hat{x}}_{k}={\hat{x}}_{k}^{s},\\ P_{k}={\bar{P}}^{s}; \end{array}$$17:     **else**18:        Smart sensor does not transmit ${\hat{x}}_{k}^{s}$, i.e., $\gamma_{k}=0$;19:        Smart sensor and estimator compute a new optimal time-triggered scheduling for time interval $\left[k+1,\;T\right]$ with *d* available communication resources and obtain a new *S*, respectively;20:        Estimator updates estimate and covariance by
$$\begin{array}{l} {\hat{x}}_{k}=A{\hat{x}}_{k-1}+K_{k}\left(b-CA{\hat{x}}_{k-1}\right),\\ P_{k}=\left(I_{n}-K_{k}C\right)h\left(P_{k-1}\right),\\ K_{k}=h\left(P_{k-1}\right)C^{⊤}{\left(Ch\left(P_{k-1}\right)C^{⊤}+R+Y^{-1}\right)}^{-1}; \end{array}$$21:     **end if**22:   **end if**23:**end for**

**Figure 5:**
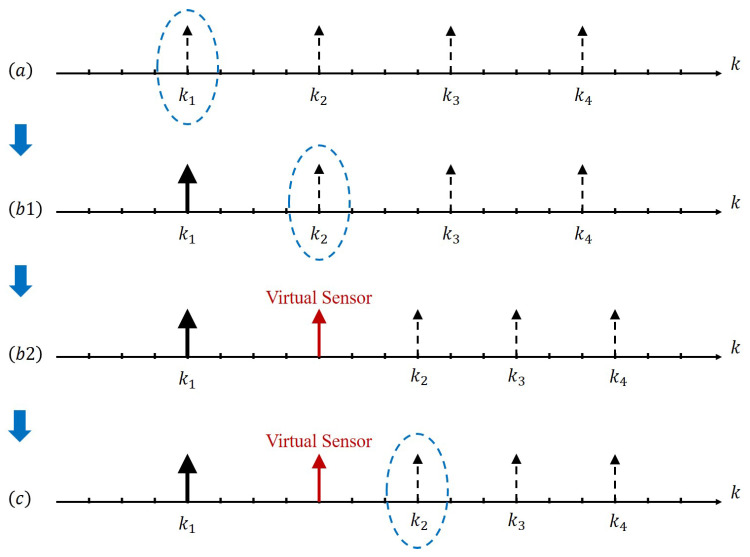
A realization under the proposed hybrid scheduling for T=20 and d=4. (**a**) The optimal time-triggered scheduling is uniformly distributed. When $k=k_{1}$, check the event triggering condition. (**b1**) The triggering condition is not satisfied, and the smart sensor transmits ${\hat{x}}_{k_{1}}^{s}$. When $k=k_{2}$, again check the event triggering condition. (**b2**) The triggering condition is satisfied, and a virtual sensor transmission happens. One real transmission chance is saved. The new optimal time-triggered scheduling is obtained. (**c**) Check the event triggering condition for the following $k_{i}$’s.

The illustration of Algorithm 1 can be found in Figure 5. The key idea is stated as follows. The smart sensor first follows the optimal time-triggered scheduling and checks the triggering condition at each scheduled transmission time. Once the event trigger works, the transmission of this time is saved for future use, while the remote estimator is equivalent to receiving a measurement from a virtual sensor. Since one chance of transmission is saved, the remaining transmission times are re-scheduled according to the optimal time-triggered scheduling within the remaining time duration.

### 3.4. Performance Analysis

In this subsection, we aim to analyze the estimation performance induced by the proposed time- and event-triggered scheduling.

We first define two operators, $\tilde{g}:S_{+}^{n}\mapsto S_{+}^{n}$ and $r:S_{+}^{n}\mapsto S_{+}^{n}$, as

(33) $$\begin{array}{cc} \tilde{g}\left(X\right) & ≜X-XC^{⊤}{\left(CXC^{⊤}+R+Y^{-1}\right)}^{-1}CX, \end{array}$$

(34) $$\begin{array}{cc} r(X) & ≜{\bar{P}}^{s}. \end{array}$$

When the smart sensor transmits ${\hat{x}}_{k}^{s}$, we have $P_{k}=r\left(P_{k-1}\right)$; when the sensor does not transmit for $k\in \left\{k_{1},k_{2},\cdots ,k_{d}\right\}$, we have $P_{k}=\tilde{g}\circ h\left(P_{k-1}\right)$; otherwise, we have $P_{k}=h\left(P_{k-1}\right)$. During the finite time horizon [1,T], the state estimation error covariance at time *t* is the result brought by *t* consecutive operators, including *r*, $\tilde{g}\circ h$, and *h*. For example, $P_{T}$ is induced by *d* times the operator *r*, $d_{1}$ times the operator $\tilde{g}\circ h$, and $d_{2}$ times the operator *h*, where $d+d_{1}+d_{2}=T$. The order of these operators is determined by the implemented scheduling policy. The upper bound of the estimation error covariance is provided as follows.

**Theorem 2.**  *With the time- and event-triggered scheduling proposed in Algorithm 1, the average state estimation error covariance during a finite time horizon, T, is upper bounded by*

(35) $$\begin{array}{c} \bar{J}=\frac{1}{T}\left(d\mathrm{Tr}\left\{{\bar{P}}^{s}\right\}+\sum_{i=1}^{T-d}\mathrm{Tr}\left\{h^{i}\left({\bar{P}}^{s}\right)\right\}\right). \end{array}$$

**Proof of Theorem 2.**  First, for the affine operator *h*, we know the following property [25]:

$$\begin{array}{c} h(X)⪰h(Y),\;\;\;\forall X⪰Y⪰0. \end{array}$$

For the operator $\tilde{g}\circ h$, we have

(36) $$\begin{array}{c} \tilde{g}\circ h\left(X\right)=h\left(X\right)-h\left(X\right)C^{⊤}{\left(Ch\left(X\right)C^{⊤}+R+Y^{-1}\right)}^{-1}Ch\left(X\right)⪯h\left(X\right),\;\;\;\forall X⪰0 \end{array}$$

and $\tilde{g}\circ h\left(X\right)⪰0$ by the error covariance definition.

Then, we consider the state estimation error covariance at the terminal time, which is induced by *T* operators, as follows

$$\begin{array}{c} P_{T}=\varphi_{T}\circ \varphi_{T-1}\circ \cdots \circ \varphi_{1}\left({\bar{P}}^{s}\right), \end{array}$$

where $\varphi_{i}\in \left\{r,\tilde{g}\circ h,h\right\},\;1\leq i\leq T$. We revisit the operators from $\varphi_{T}$ to $\varphi_{1}$. We begin with the terminal operator, $\varphi_{T}$. When $\varphi_{T}=\tilde{g}\circ h$, for given $\varphi_{T-1}^{\prime },\varphi_{T-2}^{\prime },\cdots ,\varphi_{1}^{\prime }$, there exists $\varphi_{T}\circ \varphi_{T-1}^{\prime }\circ \cdots \circ \varphi_{1}^{\prime }\left({\bar{P}}^{s}\right)⪯h\circ \varphi_{T-1}^{\prime }\circ \cdots \circ \varphi_{1}^{\prime }\left({\bar{P}}^{s}\right)$ according to the inequality (36). Then, we go through the operators in the order $\varphi_{T},\varphi_{T-1},\cdots ,\varphi_{1}$. Whenever we find an operator $\varphi_{i}=\tilde{g}\circ h$, we replace it by *h* and obtain a new operator sequence, which consists of T−d times *h* and *d* times *r*. Since h(X) is non-decreasing and affine in *X*, the new operator sequence results in a larger average state estimation error covariance, *J*. Furthermore, based on Theorem 3.1 in [26], T−d times grouped *h* would maximize *J*. As a consequence, *J* is upper bounded by $\bar{J}$, which is caused by T−d times the consecutive operator *h* and *d* times the operator *r*. □

**Remark 4.**  *Note that the upper bound $\bar{J}$ will not be achieved with the proposed time- and event-triggered scheduling in Algorithm 1. Even in the worst case, the hybrid scheduling would lead to a better estimation performance.*

Moreover, we also investigate the communication rate. Since the real transmissions always happen *d* times during the time horizon [1,T], the communication rate for the whole time duration is $\frac{d}{T}$. As for each time instance $k\in \left\{k_{1},k_{2},\ldots ,k_{d}\right\}$, the probability of transmitting ${\hat{x}}_{k}^{s}$ is provided in the following lemma. It is considered for stable systems, i.e., ρ(A)<1, and we assume that the system has entered the steady state. Then, we have $x_{k}∼N\left(0,\;\Sigma_{x}\right)$ and $y_{k}∼N\left(0,\;\Sigma_{y}\right)$, where $\Sigma_{x}=A\Sigma_{x}A^{⊤}+Q$ and $\Sigma_{y}=C\Sigma_{x}C^{⊤}+R≻0$.

**Lemma 3.**  *When the system is stable, the probability of transmitting ${\hat{x}}_{k}^{s}$ for $k\in \left\{k_{1},k_{2},\ldots ,k_{d}\right\}$ is*

$$\begin{array}{c} 1-\frac{1}{\sqrt{\det\left(I_{m}+\Sigma_{y}Y\right)}}\exp\left(-\frac{1}{2}b^{⊤}{\left(\Sigma_{y}+Y^{-1}\right)}^{-1}b\right). \end{array}$$

**Proof of Lemma 3.**  From the linearity of the system, $y_{k}$ is Gaussian distributed with a mean of zero and a covariance of $\Sigma_{y}$. From the triggering condition, we have

$$\begin{array}{cc} \mathrm{Pr}\left(\gamma_{k}=1\right) & =\mathrm{Pr}\left(\zeta_{k}>\phi \left(y_{k}\right)\right)\\ \; & =1-E\left[\exp\left(-\frac{1}{2}{\left(y_{k}-b\right)}^{⊤}Y\left(y_{k}-b\right)\right)\right]\\ \; & =1-\int_{R^{m}}\frac{1}{\sqrt{\det\left(\Sigma_{y}\right){\left(2\pi \right)}^{m}}}\exp\left(-\frac{1}{2}\left[{\left(y_{k}-b\right)}^{⊤}Y\left(y_{k}-b\right)+y_{k}^{⊤}\Sigma_{y}^{-1}y_{k}\right]\right)dy_{k}. \end{array}$$

By completing the square, we have

$$\begin{array}{cc} \; & {\left(y_{k}-b\right)}^{⊤}Y\left(y_{k}-b\right)+y_{k}^{⊤}\Sigma_{y}^{-1}y_{k}\\ =\; & {\left[y_{k}-{\left(\Sigma_{y}^{-1}+Y\right)}^{-1}Yb\right]}^{⊤}\left(\Sigma_{y}^{-1}+Y\right)\left[y_{k}-{\left(\Sigma_{y}^{-1}+Y\right)}^{-1}Yb\right]+b^{⊤}{\left(\Sigma_{y}+Y^{-1}\right)}^{-1}b, \end{array}$$

where the equality holds based on the matrix inversion lemma. Since the integral of a Gaussian probability density function equals one, we obtain

$$\begin{array}{cc} \mathrm{Pr}\left(\gamma_{k}=1\right) & =1-\frac{\sqrt{\det\left({\left(\Sigma_{y}^{-1}+Y\right)}^{-1}\right)}}{\sqrt{\det\left(\Sigma_{y}\right)}}\exp\left(-\frac{1}{2}b^{⊤}{\left(\Sigma_{y}+Y^{-1}\right)}^{-1}b\right)\\ \; & =1-\frac{1}{\sqrt{\det\left(I_{m}+\Sigma_{y}Y\right)}}\exp\left(-\frac{1}{2}b^{⊤}{\left(\Sigma_{y}+Y^{-1}\right)}^{-1}b\right), \end{array}$$

which concludes the proof. □

## 4. Simulation Examples

In this section, we take the linearized discrete-time model of a simplified longitudinal flight system (see [27] for more details) as an example to illustrate the effectiveness of the proposed time- and event-triggered hybrid scheduling. The state variable $x_{k}\in R^{3}$ indicates the pitch angle, the pitch rate, and the normal velocity. The system parameters are as follows:

$$\begin{array}{c} A=\left[\begin{array}{ccc} 0.99 & -0.12 & -0.43\\ 0 & 0.99 & -0.07\\ 0 & 0.82 & 0 \end{array}\right],\;\;C=\left[\begin{array}{ccc} 1 & 0 & 0 \end{array}\right],\;\;Q=\left[\begin{array}{ccc} 0.01 & 0 & 0\\ 0 & 0.01 & 0\\ 0 & 0 & 0.0001 \end{array}\right],\;\;R=0.01. \end{array}$$

**Figure 6:**
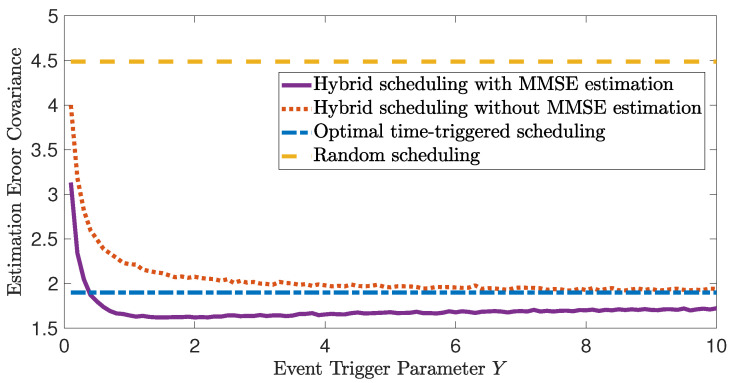
Illustration of the effectiveness of the hybrid scheduling and MMSE estimation.

The initial value $\Pi_{0}=0.01I_{3}$. We set T=300 and the available communication chances as d=10. Then, from the optimal time-triggered scheduling in Lemma 1, one transmission time index sequence is $\left\{27,54,81,108,135,162,189,217,245,273\right\}$. The associated average state estimation error covariance trace is J=1.8998. As a comparison, we calculate the average estimation error covariance of the random scheduling, which randomly chooses 10 time instances to transmit ${\hat{x}}_{k}^{s}$ within the interval [1,300]. The result is J=4.4881. To examine the performance of the proposed hybrid scheduling (Algorithm 1), we set b=0.1 and let *Y* take a value in [0.1,10]. The average state estimation error covariance trace with respect to the event trigger parameter *Y* is depicted in Figure 6 with a purple line. As a comparison, the state estimation of the situation where only the time updates (18) and (19) are performed when $\zeta_{k}\leq \phi \left(y_{k}\right)$ is plotted with a red dotted line. Since the purple line is generated through the utilization of the proposed MMSE state estimation, it consistently demonstrates superior performance compared to the identical hybrid scheduling approach without MMSE estimation. Note that all of the above-mentioned curves are obtained by running 10,000 simulations. It can be found that when Y=1.5, the proposed hybrid scheduling achieves the best performance, with J=1.6203. In addition, with the increase in *Y*, the estimation error covariance of the proposed hybrid scheduling decreases first and then approaches the optimal time-triggered scheduling. This is because when *Y* is small, the result of the triggering condition (12) is always $\gamma_{k}=0$. Then, situations where all of the communication chances are saved until the end of the time interval may happen, which will induce a worse estimation performance compared with the optimal time-triggered scheduling. When *Y* is large enough, the result of the triggering condition (12) is more likely to be $\gamma_{k}=1$, which is exactly the optimal time-triggered scheduling. When *Y* is chosen appropriately, the event trigger works and saves the communication resource when the estimation performance is good. The precious transmission chance is allocated for the remaining time interval. Note that the performance upper bound is $\bar{J}=16.8301$.

## 5. Conclusions and Future Work

In this paper, we investigated a sensor scheduling problem for the remote state estimation with limited communication resources. Based on the existing optimal time-triggered scheduling, a stochastic event trigger was provided. The MMSE state estimate was derived since the Gaussian property was preserved. Hence, we proposed a hybrid scheduling approach. The estimation performance at the remote estimator was enhanced compared with the time-triggered scheduling under the same communication rate.

With the purpose of extending the finite-time-horizon result, one possible future direction is to investigate the infinite-time-horizon optimization problem with a limited communication rate. Additionally, it would be interesting to investigate the design of the parameters of the event trigger. Parameter optimization problems could be formulated to achieve a better state estimation performance.

## Data Availability

Not applicable.

## Appendix A. Proof of Lemma 2

Define a block matrix $\Delta =\left[\begin{array}{cc} \Delta_{xx} & \Delta_{xy}\\ \Delta_{yx} & \Delta_{yy} \end{array}\right]\in S_{++}^{n+m}$ such that $\Delta =\Phi^{-1}$, where $\Delta_{xx}\in S_{++}^{n}$, $\Delta_{yy}\in S_{++}^{m}$, and $\Delta_{xy}=\Delta_{yx}^{⊤}$. Then, we have

$$\begin{array}{c} \Theta ={\left[\begin{array}{cc} \Delta_{xx} & \Delta_{xy}\\ \Delta_{yx} & \Delta_{yy}+Y \end{array}\right]}^{-1}. \end{array}$$

Furthermore, there is

(A1) $$\begin{array}{cc} \Theta & ={\left(\left[\begin{array}{cc} I_{n} & 0\\ \Delta_{yx}\Delta_{xx}^{-1} & I_{m} \end{array}\right]\left[\begin{array}{cc} \Delta_{xx} & 0\\ 0 & \Delta_{yy}+Y-\Delta_{yx}\Delta_{xx}^{-1}\Delta_{xy} \end{array}\right]\left[\begin{array}{cc} I_{n} & \Delta_{xx}^{-1}\Delta_{xy}\\ 0 & I_{m} \end{array}\right]\right)}^{-1}\\ \; & =\left[\begin{array}{cc} I_{n} & -\Delta_{xx}^{-1}\Delta_{xy}\\ 0 & I_{m} \end{array}\right]\left[\begin{array}{cc} \Delta_{xx}^{-1} & 0\\ 0 & {\left(\Delta_{yy}+Y-\Delta_{yx}\Delta_{xx}^{-1}\Delta_{xy}\right)}^{-1} \end{array}\right]\left[\begin{array}{cc} I_{n} & 0\\ -\Delta_{yx}\Delta_{xx}^{-1} & I_{m} \end{array}\right]. \end{array}$$

By calculating the product of the above three matrices, we have

(A2) $$\begin{array}{cc} \Theta_{xx} & =\Delta_{xx}^{-1}+\Delta_{xx}^{-1}\Delta_{xy}{\left(\Delta_{yy}+Y-\Delta_{yx}\Delta_{xx}^{-1}\Delta_{xy}\right)}^{-1}\Delta_{yx}\Delta_{xx}^{-1}, \end{array}$$

(A3) $$\begin{array}{cc} \Theta_{xy} & =-\Delta_{xx}^{-1}\Delta_{xy}{\left(\Delta_{yy}+Y-\Delta_{yx}\Delta_{xx}^{-1}\Delta_{xy}\right)}^{-1}, \end{array}$$

(A4) $$\begin{array}{cc} \Theta_{yy} & ={\left(\Delta_{yy}+Y-\Delta_{yx}\Delta_{xx}^{-1}\Delta_{xy}\right)}^{-1}. \end{array}$$

Similar to the decomposition procedure (A1) for $\Phi =\Delta^{-1}$, we have

(A5) $$\begin{array}{cc} \Phi_{yy} & ={\left(\Delta_{yy}-\Delta_{yx}\Delta_{xx}^{-1}\Delta_{xy}\right)}^{-1}. \end{array}$$

Combining Equations (A4) and (A5), we obtain

(A6) $$\begin{array}{c} \Theta_{yy}={\left(\Phi_{yy}^{-1}+Y\right)}^{-1}. \end{array}$$

Since $\Delta \Phi =I_{n+m}$, we have $\Delta_{xx}\Phi_{xy}+\Delta_{xy}\Phi_{yy}=0$, which implies $-\Delta_{xx}^{-1}\Delta_{xy}=\Phi_{xy}\Phi_{yy}^{-1}$. Hence, for Equation (A3), we have

(A7) $$\begin{array}{c} \Theta_{xy}=\Phi_{xy}\Phi_{yy}^{-1}{\left(\Phi_{yy}^{-1}+Y\right)}^{-1}=\Phi_{xy}{\left(I_{m}+Y\Phi_{yy}\right)}^{-1}. \end{array}$$

Again, $\Delta =\Phi^{-1}$ can be decomposed as follows:

(A8) $$\begin{array}{cc} \Delta & ={\left(\left[\begin{array}{cc} I_{n} & \Phi_{xy}\Phi_{yy}^{-1}\\ 0 & I_{m} \end{array}\right]\left[\begin{array}{cc} \Phi_{xx}-\Phi_{xy}\Phi_{yy}^{-1}\Phi_{yx} & 0\\ 0 & \Phi_{yy} \end{array}\right]\left[\begin{array}{cc} I_{n} & 0\\ \Phi_{yy}^{-1}\Phi_{yx} & I_{m} \end{array}\right]\right)}^{-1}\\ \; & =\left[\begin{array}{cc} I_{n} & 0\\ -\Phi_{yy}^{-1}\Phi_{yx} & I_{m} \end{array}\right]\left[\begin{array}{cc} {\left(\Phi_{xx}-\Phi_{xy}\Phi_{yy}^{-1}\Phi_{yx}\right)}^{-1} & 0\\ 0 & \Phi_{yy}^{-1} \end{array}\right]\left[\begin{array}{cc} I_{n} & -\Phi_{xy}\Phi_{yy}^{-1}\\ 0 & I_{m} \end{array}\right]. \end{array}$$

By computing the product of the above three matrices, we obtain

(A9) $$\begin{array}{c} \Delta_{xx}={\left(\phi_{xx}-\Phi_{xy}\Phi_{yy}^{-1}\Phi_{yx}\right)}^{-1}. \end{array}$$

Then, for $\Theta_{xx}$ in Equation (A2), we can further derive

(A10) $$\begin{array}{cc} \Theta_{xx} & =\phi_{xx}-\Phi_{xy}\Phi_{yy}^{-1}\Phi_{yx}+\Delta_{xx}^{-1}\Delta_{xy}{\left(\Delta_{yy}+Y-\Delta_{yx}\Delta_{xx}^{-1}\Delta_{xy}\right)}^{-1}\Delta_{yx}\Delta_{xx}^{-1}\\ \; & =\phi_{xx}-\Phi_{xy}\Phi_{yy}^{-1}\Phi_{yx}+\Phi_{xy}\Phi_{yy}^{-1}{\left(\Phi_{yy}^{-1}+Y\right)}^{-1}\Phi_{yy}^{-1}\Phi_{yx}\\ \; & =\phi_{xx}-\Phi_{xy}{\left(\Phi_{yy}+Y^{-1}\right)}^{-1}\Phi_{yx}, \end{array}$$

where the second equality holds because of $\Delta \Phi =I_{n+m}$ and Equations (A4) and (A6). The third equality results from

$$\begin{array}{c} {\left(\Phi_{yy}^{-1}+Y\right)}^{-1}=\Phi_{yy}-\Phi_{yy}{\left(\Phi_{yy}+Y^{-1}\right)}^{-1}\Phi_{yy}, \end{array}$$

which is directly induced by the matrix inversion lemma.

Finally, Equations (A6), (A7) and (A10) are all derived, and the proof is finished.
